# Inhibition of Metabolic Shift can Decrease Therapy Resistance in Human High-Grade Glioma Cells

**DOI:** 10.1007/s12253-019-00677-2

**Published:** 2019-06-11

**Authors:** Gábor Petővári, Titanilla Dankó, Ildikó Krencz, Zoltán Hujber, Hajnalka Rajnai, Enikő Vetlényi, Regina Raffay, Judit Pápay, András Jeney, Anna Sebestyén

**Affiliations:** 1st Department of Pathology and Experimental Cancer Research, Semmelweis University, Üllői út 26, Budapest, H-1085 Hungary

**Keywords:** Glioma, Metabolism, Metabolic shift, mTORC2, Combination therapy

## Abstract

The high-grade brain malignancy, glioblastoma multiforme (GBM), is one of the most aggressive tumours in central nervous system. The developing resistance against recent therapies and the recurrence rate of GBMs are extremely high. In spite several new ongoing trials, GBM therapies could not significantly increase the survival rate of the patients as significantly. The presence of inter- and intra-tumoral heterogeneity of GBMs arise the problem to find both the pre-existing potential resistant clones and the cellular processes which promote the adaptation mechanisms such as multidrug resistance, stem cell-ness or metabolic alterations, etc. In our work, the in situ metabolic heterogeneity of high-grade human glioblastoma cases were analysed by immunohistochemistry using tissue-microarray. The potential importance of the detected metabolic heterogeneity was tested in three glioma cell lines (grade III-IV) using protein expression analyses (Western blot and WES Simple) and therapeutic drug (temozolomide), metabolic inhibitor treatments (including glutaminase inhibitor) to compare the effects of rapamycin (RAPA) and glutaminase inhibitor combinations in vitro (Alamar Blue and SRB tests). The importance of individual differences and metabolic alterations were observed in mono-therapeutic failures, especially the enhanced Rictor expressions after different mono-treatments in correlation to lower sensitivity (temozolomide, doxycycline, etomoxir, BPTES). RAPA combinations with other metabolic inhibitors were the best strategies except for RAPA+glutaminase inhibitor. These observations underline the importance of multi-targeting metabolic pathways. Finally, our data suggest that the detected metabolic heterogeneity (the high mTORC2 complex activity, enhanced expression of Rictor, p-Akt, p-S6, CPT1A, and LDHA enzymes in glioma cases) and the microenvironmental or treatment induced metabolic shift can be potential targets in combination therapy. Therefore, it should be considered to map tissue heterogeneity and alterations with several cellular metabolism markers in biopsy materials after applying recently available or new treatments.

## Introduction

The high-grade glial tumour, glioblastoma multiforme (GBM), is one of the most aggressive and invasive tumours in central nervous system. Overall median survival of patients is approximately 8 months after GBM is diagnosed. The current standard therapy is surgical resection followed by adjuvant radiotherapy and/or chemotherapy.

Many years ago, temozolomide (TMZ) was introduced. TMZ administration could increase the survival moderately (up to 1 year) in trials for recurrent GBM to improve patients’ survival [1, 2]. The developing resistance against TMZ (nearly 100% in all available treatment combinations) and the recurrence rate of GBMs are extremely high. The combined therapies (radiochemotherapy or other targeted therapy combinations) have more success and can increase the survival time and rate. Applying novel combined therapies, 2-year survival can be achieved in 27% of the cases comparing to the effect of radiotherapy or TMZ treatment alone (10%). However, GBM is still an incurable disease [3, 4]. Several novel trials are ongoing including VEGF inhibitor alone or in combinations. Moreover, in case of certain immunotherapies, with limitations and strict requirements – i.e. the drugs have to be transported across the blood-brain barrier, phase III trials are about to start [5, 6]. Despite there have been large developments in targeted therapy research, the currently available therapies could not increase the survival rate of GBM patients as significantly as it is observed in other solid tumours [7].

Recent studies highlighted that tumoral heterogeneity and different adaptation mechanisms can promote tumour evolution in high-grade malignancies. The presence of inter- and intra-tumoral heterogeneity of GBMs was described using many techniques including genetic studies and radiological imaging approaches [8–10]. These and other (e.g. microenvironmental, metabolic) types of tumoral heterogeneity raise the problem of finding both the pre-existing potential resistant clones and the cellular processes which promote the adaptation mechanisms such as multidrug resistance, stem cell-ness or metabolic alterations, etc. [11].

To design effective (novel) therapies the characteristics of intra-tumoral heterogeneity and the adaptation mechanisms during resistance evolution and/or relapsed tumour development are needed to study in details [9]. Tumour cells have high adaptation capacity using many different strategies including metabolic shifts. In tumour tissues, cells with different characteristics are to find the way to fuel energy and macromolecule production, and additionally to develop a resistant state in which the survival and growth are assured even in highly toxic microenvironment [12]. As for this adaptation, the surviving and proliferating cells need to orchestrate the speed of TCA cycle, oxidative phosphorylation (OXPHOS), pentose-phosphate pathway, amino-acid and lipid synthesis etc. The oncogenic, tumorigenic alterations influence certain processes including glycolysis, glutaminolysis, mitochondrial oxidative function, lipid metabolism and autophagy [13]. To satisfy these needs certain metabolic shifts can be proceeded at cellular level in tumour cells, e.g. alterations in the expression and the activity of several metabolic enzymes and proteins [14].

**Fig. 1:**
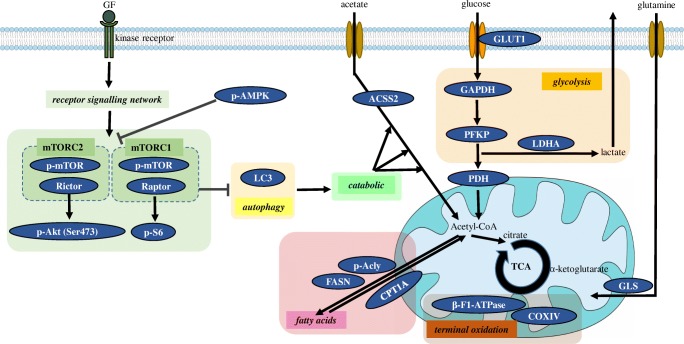
Schematic figure of the studied metabolic pathways. The expressions of glutaminolysis (GLS), glycolysis (GLUT1, GAPDH, PFKP LDHA, PDH), fatty acid metabolism (FASN, p-Acly, CPT1A), acetate consumption (ACSS2), mitochondrial oxidative phosphorylation (COXIV, β-F1-ATPase), autophagy (LC3), ATP sensor protein (p-AMPK) and mTOR complex activity (p-mTOR, Raptor, Rictor, p-S6, p-Akt (Ser473)) related enzymes were studied by Western blot analyses, WES Simple. Further explanation can be found in the text

Cellular metabolism can be characterised by mapping the expression and activity of different TCA cycle, glycolytic enzymes, transporters and kinases (Fig. 1.).

An important pathway in metabolic plasticity is the mammalian target of rapamycin (mTOR) centred signalling regulatory network. mTOR hyperactivation is characteristic for several tumours even for GBMs [15]. However, its regulatory role depends on different complex formation and activity; these complexes differ in their core proteins, targets, functions (including metabolic functions) and inhibitor sensitivities. Phospho-mTOR (in both mTORC1 and C2 complex activity could correlate to the amount of p-mTOR), Raptor (a characteristic protein for mTORC1), Rictor (a characteristic protein for mTORC2) are the main markers for characterising the amount and activity of mTOR. Additionally, phospho-S6 and phospho-(Ser473)-Akt (the activated targets of mTORC1 and C2, respectively) could also be used to complete the study of mTOR complexes activity [16].

The growth regulatory role of mTOR is well-known; it has many metabolic effects – directly influencing the protein synthesis, lipid metabolism and autophagy besides it indirectly influences many different genes (CPT1A – carnitine O-palmitoyltransferase 1, GLS - glutaminase, c-myc, etc.), which alter cellular metabolic activity [17].

Glycolytic activity, Warburg effect and mitochondrial metabolic functions can be monitored by the expression changes of e.g. glucose transporter 1 (GLUT1), phosphofructokinase (PFKP), glyceraldehyde-3-phosphate dehydrogenase (GAPDH), pyruvate dehydrogenase (PDH), lactate dehydrogenase (LDH) and ATP synthase subunit beta (β-F1-ATPase), cytochrome c oxidase subunit 4 (COXIV) [13, 18]. Lipid synthesis, oxidation and their ratio can also be followed by studying the expression of fatty acid synthase (FASN), ATP-citrate synthase (p-Acly) and CPT1A [19]. Moreover, the bioenergetic states, the energy supply level can be characterised by phospho-AMP-activated kinase (p-AMPK), autophagy can be monitored by the expression of microtubule-associated protein 1A/1B-light chain 3 (LC3) [20]. Glutamine and other possible alternative substrate consumption rate can be analysed by GLS, Acyl-coenzyme A synthetase short-chain family member 2 (ACSS2) levels [21], as well. The potential plasticity of substrate utilisation plasticity could also be interesting in brain tumours and brain metastases [14].

Many different compounds, including different TMZ combinations have been tested in glioma models in vitro and in vivo, however, the expected metabolic plasticity, the alterations in metabolic enzyme expression and activity have been less studied in high-grade gliomas. In our work, we analysed the in situ metabolic heterogeneity of high-grade (III-IV) isocitrate dehydrogenase (IDH) wild-type human glioblastoma cases using immunohistochemistry study. To understand the potential importance of the detected metabolic heterogeneity, we studied three IDH wild-type (grade III-IV) glioma cell lines further. TMZ, rapamycin (mTOR inhibitor) and several metabolic or other inhibitors (chloroquine, autophagy inhibitor; glutaminase inhibitor, etomoxir – lipid oxidation inhibitor, doxycycline – antibiotics with potential metabolic/mitochondrial inhibitory effect) were tested to analyse the alterations both in cellular proliferation and metabolic enzyme expression/activity after in vitro treatments. Finally, we studied the potential anti-proliferative effects of combined inhibitors in vitro for sensitising tumour cells to the used agents/treatments.

## Materials and Methods

### All Materials were Purchased from Merck-Sigma-Aldrich, except where it is Indicated in the Text

Human glioma cell lines – U373 Uppsala (U373-U; ECACC-08061901), U251 and U87 (ECACC-09063001, with PTEN, NF-1, p53 and MSH2 mutations; and ATCC-HTB-14, characteristic mutations in PTEN, NF-1 and Notch-2, respectively) were maintained in DMEM high glucose medium (Biosera) supplemented with 10% foetal bovine serum (FBS; Biosera), 2 mM L-glutamine (Biosera) and 100 UI/ml penicillin–streptomycin (Biosera) at 37 °C with 5% CO_2_. After seeding, the cells were treated with different drugs (rapamycin-RAPA, 50 ng/ml; doxycycline-DOXY, 10 μM; temozolomide-TMZ, 100 μM; etomoxir-ETO, 50 μM; chloroquine-CHL, 50 μM and bis-2-(5-phenylacetoamido-1,3,4- thiadiazol-2-yl)-ethyl sulfide- BPTES, 10 μM) and their combinations in 96-well plates (2–5 × 10^3^ cells/well for proliferation tests) or in T25 flasks (3–6 × 10^5^ cells/flask – for Western blot experiments) for 72 h. The concentration of the drugs was applied based on our previous publications [22]. Lower dose than IC50 level was applied in drug combinations, these concentrations were defined in accordance with previously published data on IC50 [23–26]. The anti-proliferative effects of the treatments were measured after a 4-h incubation period using Alamar Blue (Thermo Fisher Scientific). The fluorescence was measured at 570–590 nm (Fluoroskan Ascent FL fluorimeter; Labsystems International) and the results were analysed by Ascent Software. To detect the protein content related growth inhibitory effect, SRB test was performed as the following: after 10% trichloroacetic acid fixation, the cells were incubated with sulforhodamine B (15 min, 0.4 m/v%) then 10 mM Tris base was added to each well to solubilise the protein-bound dye. The absorbance was measured at 570 nm in a microplate reader. At least 3 independent experiments were performed with 6 parallels in each. Percentage of the cell proliferation was given relative to control samples. To analyse the additive or synergistic effects of different drug combinations the Combination Index (CI) was calculated as we described previously [22].

### mTOR and Metabolic Protein Expression Analysis by Western Blot and WES Simple Capillary Immunoassay

Proteins were extracted (using 50 mM Tris, 10% glycerol, 150 mM NaCl, 1% Nonidet-P40, 10 mM NaF, 1 mM phenylmethylsulfonyl fluoride, 0.5 mM NaVO_3_, pH 7.5) from 1 × 10^6^ cells. The protein lysates were quantitated by Bradford reagent (BioRad). Sodium dodecyl sulfate polyacrylamide gel electrophoresis and PVDF membranes (BioRad) were used in Western blot analysis. The applied primary antibodies were listed in Table 1.; HRP-conjugated anti-β-actin (1:10000; ab49900, Abcam) was used as loading control. At the end, biotinylated secondary antibodies and avidin-HRP complex (Vectastain Elite ABC Kit, Vector), enhanced chemiluminescence technique (Pierce ECL Western Blotting Substrate) with Li-Cor-C-Digit photo documentation system were applied. Image Studio Digits program was used to perform densitometric analysis.

**Table 1:** List of the used primary antibodies (catalogue numbers, dilutions and expected sizes for Western blot analyses were given)

Primary antibody	IHC		Western blot		WES		size (kDa)
	Cat. No.	Dilution	Cat. No.	Dilution	Cat. No.	Dilution	
p-mTOR	CST #2976	1:100	CST #2971	1:1000	CST #2971	1:50	289
Rictor	Bethyl A500-002A	1:1000	CST #2140	1:1000	CST #2140	1:50	200
Raptor	–	–	Abcam 40,768	1:1000	Abcam 40,768	1:50	150
p-Akt (Ser473)	Novus 79,891	1:50	CST #4060	1:2000	CST #4060	1:50	60
p-S6	CST #2211	1:100	CST #4858	1:1000	CST #4858	1:50	32
ACSS2	–	–	–	–	CST #3658	1:50	78
FASN	CST #3180	1:50	–	–	CST #3180	1:50	273
p-Acly	–	–	CST #4331	1:1000	CST #4331	1:50	125
CPT1A	Abcam 128,568	1:500	Abcam 128,568	1:1000	Abcam 12,8568	1:50	88
GLS	Abcam 156,876	1:200	Abcam 156,876	1:1000	Abcam 15,6876	1:50	65
GLUT1	–	–	Abcam 652	1:500	–	–	45–60
PFKP	–	–	CST #8164	1:1000	–	–	80
GAPDH	–	–	Abcam 8245	1:10000	Abcam 8245	1:50	37
PDH	–	–	CST #3205	1:1000	CST #3205	1:50	43
LDHA	CST #3582	1:400	CST #3582	1:1000	CST #3582	1:50	37
β-F1-ATPase	Abcam 14,730	1:100	Abcam 14,730	1:2000	–	–	52
COXIV	–	–	–	–	CST #4850	1:50	17
p-AMPK (Thr172)	–	–	CST #2535	1:1000	CST #2535	1:50	62
LC3	–	–	–	–	Novus 110–57179	1:50	14–16
β-actin	–	–	Abcam 49,900	10000	Sigma A2228	1:50	45

WES Simple analysis was performed on WES system (ProteinSimple-Biotechne 004–600) according to the manufacturer’s instructions. 12–230 kDa Separation Module (ProteinSimple SM-W004) and either the Anti-Rabbit Detection Module (ProteinSimple DM-001), Anti-Mouse Detection Module (ProteinSimple DM-002) or Anti-mouse IgG, HRP-linked Antibody (Cell Signaling Technologies, CST #7076) were applied depending on the primary antibodies. In brief, glioma cell samples were diluted to an appropriate concentration (0.2 or 1 μg/μl depending on the secondary antibody used) in sample buffer (100x diluted ‘10x Sample Buffer’ from the Separation Module), then mixed with Fluorescent Master Mix 1:4 and heated at 95 °C for 5 min. The samples, the blocking reagent (antibody diluent), the primary antibodies, the HRP-conjugated secondary antibodies and the chemiluminescent substrate were added to the plate. The default settings of the device were the following: stacking and separation at 395 V for 30 min; blocking reagent for 5 min, primary and secondary antibodies both for 30 min; luminol/peroxide chemiluminescence detection for 15 min (exposure times were selected for the antibodies between 1 and 512 s). The electropherograms were checked then the automatic peak detection was manually corrected if it was required. The used primary antibodies and their dilutions were given in Table 1.

### Tissue Microarray from Human High-Grade Glioma Biopsies and Immunohistochemistry Analysis

Archived tissue blocks were used with the approval of The Hungarian Scientific Council National Ethics Committee for Scientific Research (No. 7/2006). IDH wild-type human glioblastoma and high-grade astrocytoma tissues (*n* = 18) were selected for immunohistochemistry analyses. Peri-tumoral brain tissue was used as control (*n* = 2). The glioma samples were re-reviewed and reclassified according to The World Health Organization Classification of Tumors of the Central Nervous System (2016) [27]. The clinicopathological data are summarised in Table 2.

**Table 2:** Clinicopathological data of patients with high-grade glial tumour

	n = 18
Age	
< 65 years	12
≥ 65 years	6
Sex	
Male	6
Female	12
Type	
Anaplastic astrocytoma (grade III)	4
Glioblastoma (grade IV)	14
Ki67 proliferation index	
< 20%	5
≥ 20%	13

Immunohistochemistry (IHC) was performed on tissue microarrays (TMAs) with at least duplicate or triplicate cores per patient. Representative areas were selected by a neuropathologist. Antigen retrieval (pH = 6 citric acid buffer, 30 min) was performed after deparaffinisation and endogenous peroxidase blocking. Slides were incubated with primary antibodies summarised in Table 1. Sections were stained using biotin-free anti-rabbit/mouse IgG polymer-peroxidase conjugate system (Novolink, Leica). Immunoreactions were revealed using a diaminobenzidine (DAB, Dako) chromogen-hydrogen peroxide substrate. Harris haematoxylin was applied for highlighting the cell nuclei. Immunostained TMA sections were digitally scanned at 20X magnification using a Panoramic scan instrument (3D Histech) equipped with Carl Zeiss objective (NA = 0.83; Carl Zeiss MicroImaging Inc.) then analysed with CaseViewer 2.3 Software (3D Histech). Considering the intra-tumoral heterogeneity, the H-score was used to analyse the expression of mTOR and metabolism-related proteins. Based on our previously described method, H-score was calculated for each core of the TMA multiplying the fraction of tumour cell immunopositivity (%) and staining intensity scale (0, 1+, 2+ or 3+) [28]. The mean of the H-scores for each sample was calculated from the evaluated cores of the same biopsy specimens using two independent evaluations.

### Statistical Analysis

Data are presented as mean ± SD deviation. Statistical analysis was performed using IBM SPSS (version 22; SPSS Inc.) and PAST (version 3.24) software. Data evaluation of in vitro experiments was performed using Student’s t (two-tailed) test and one-way analysis of variance (ANOVA). Mann-Whitney U-test was used to determine associations between clinicopathological parameters and IHC results. Spearman correlation was used to evaluate the correlation between protein expressions. Statistical significance was defined as *p* < 0.05.

## Results

### Metabolic Heterogeneity of High-Grade Glioma Cases Based on Immunohistochemistry Analyses

**Fig. 2:**
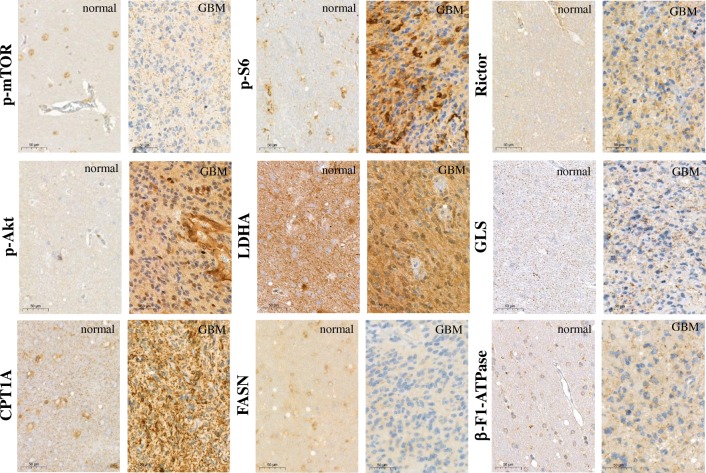
Representative immunostainings with different metabolic markers in human high-grade gliomas and normal brain tissues. The antibody-stainings were developed with DAB substrate (brown) and counterstained using haematoxylin. mTOR activity was characterised by p-mTOR, p-S6, Rictor, p-Akt stainings, other metabolic activity correlates to LDHA, GLS, CPT1A/FASN and β-F1-ATPase

Similarly, the p-mTOR expression observed in normal brain tissues with a mean H-score of 80, moderate p-mTOR staining with a mean H-score of 107 was detected in high-grade glioma samples in almost all cases (Fig. 2). In contrast, in many cases p-S6, Rictor and p-Akt protein expression patterns showed elevated mTOR, especially mTORC2 complex activities compared to normal brain tissues. The mean H-scores for p-S6, Rictor and p-Akt were 170, 128 and 190 in glioma samples and 70, 90, and 80 in normal brain tissue samples, respectively. The evaluation of other metabolic IHC stainings suggests that in the studied 18 cases high-grade glioma cells have characteristic elevated metabolic activity which correlates to a significant metabolic versatility in substrate utilisation. LDHA expression was elevated in normal brain tissues as well as in tumour samples. In addition, several substrate utilisation capacities can be observed in these glioma cells (e.g. GLS and CPT1A protein expression levels are higher in glioma cells). The lipid metabolism was shifted to catabolic β-oxidation pathway (CPT1A) from anabolic lipid synthesis (FASN). β-F1-ATPase, a marker of terminal oxidation, was also overexpressed in glioma tissues and showed a strong positive correlation to GLS expression (R = 0.753, *p* = 0.001) in glioma cells. Representative glioma and normal brain biopsy stainings show these differences (Fig. 2). Mainly, there was no association between the expression of the studied proteins and clinicopathological parameters such as age and gender. However, p-Akt expression was higher in grade IV glioblastomas than in grade III anaplastic astrocytomas. Moreover, higher FASN expression was associated with higher Ki67 proliferation index (Ki67 < 20% vs. Ki67 ≥ 20%, *p* = 0.001).

### Metabolic Diversity of Human GBM Cell Lines

**Fig. 3:**
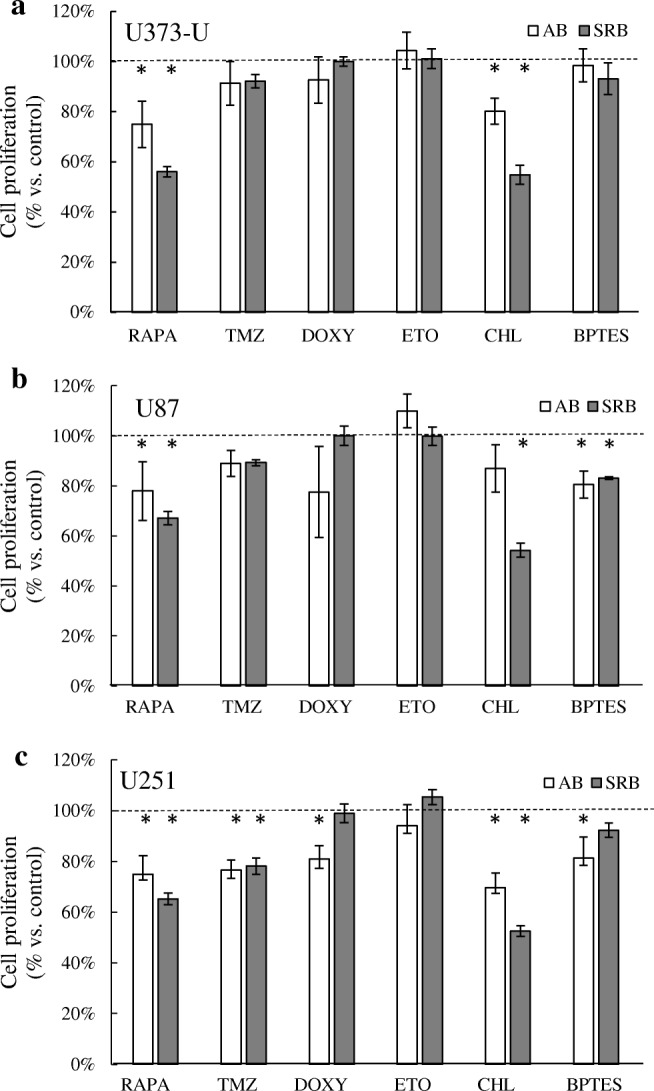
Metabolic inhibitor and TMZ sensitivity differences in human high-grade glioma cells. The anti-proliferative effects of several metabolic treatments were shown using Alamar Blue (AB) and SRB tests (RAPA – rapamycin 50 ng/ml; TMZ – temozolomide 100 μM; DOXY – doxycycline 10 μM; ETO – etomoxir 50 μM; CHL – chloroquine 50 μM; BPTES – bis-2-(5-phenylacetoamido-1,3,4- thiadiazol-2-yl)-ethyl sulfide 10 μM were applied for 72 h) in U373-U (**a**), U87 (**b**) and U251 (**c**) cells. The significant alterations were labelled * (*p* = 0.01)

**Fig. 4:**
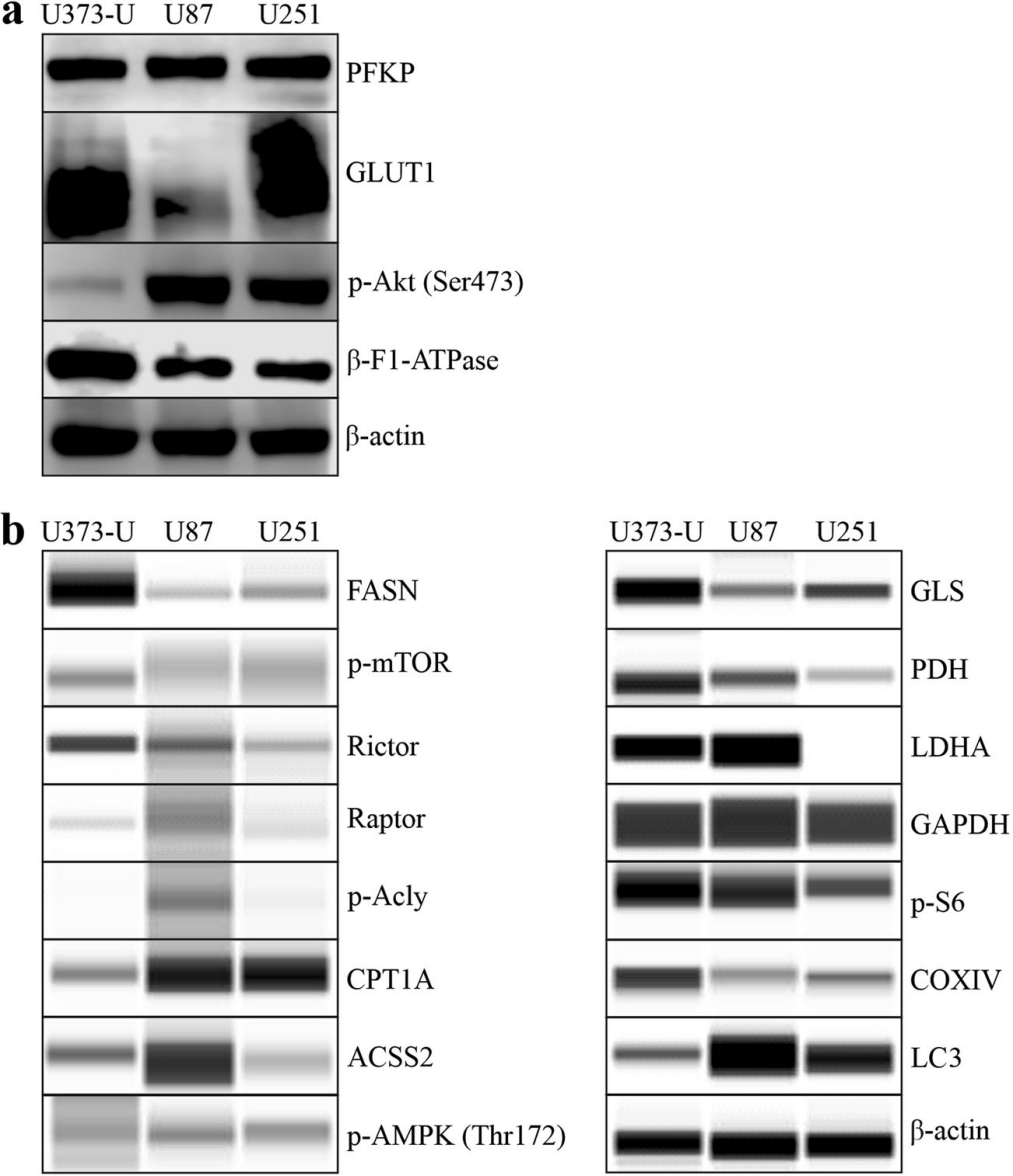
The metabolic activity related enzyme and protein expressionsin human high-grade glioma cells. The metabolic profile differences were analysed in three high-grade glioma cell lines using Western blot (**a**) or WES Simple capillary immunoassay system (**b**) - the predicted protein sizes were shown in Table 1

The characteristic mTOR inhibitor (mTORI) sensitivity of high-grade glioma cell lines has been studied previously. In our in vitro study, the proliferation could not be inhibited significantly by the common chemotherapeutic TMZ after 72-h treatment in two of the studied cell lines. However, we could confirm that the mTORC1 inhibitor RAPA significantly inhibited the proliferation of all high-grade glioma cell lines based on both Alamar Blue and SRB test results (Fig. 3). Our data are in good correlation to our other findings at protein level (using Western blot or WES Simple capillary immunoassay) in which we revealed that these cells have both mTORC1/C2 complex related high activity (detectable p-mTOR – activated form of mTOR kinase; high Rictor, p-Akt and p-S6 protein expressions) (Fig. 4). Other metabolic inhibitors have rather cell line dependent anti-proliferative effects: CHL – autophagy inhibitor could inhibit the proliferation in all studied cell lines, BPTES only in U87 and U251 cells and DOXY only in U251. Based on these, the most resistant cell line was U373-U (Fig. 3). To compare the differences, expressions of several other metabolic enzymes and proteins were studied. The protein expression profile of the three cell lines could also show some individual differences as a consequence of inter-tumoral heterogeneity. Some of these really interesting findings are the following: there is no detectable LDHA - the Warburg effect related enzyme - protein expression and the levels of β-F1-ATPase, COXIV and PDH were low in U251 and GLS expression was the lowest in U87 cells. The detected expression patterns (Fig. 4) could correlate to the sensitivity differences. Mainly, lower metabolic activity of a cell line (without glycolytic phenotype and lower mitochondrial activity) can correlate to higher inhibitor sensitivity. There are only few differences in the detected anti-proliferative alterations comparing the Alamar Blue and SRB test results, however, the SRB data were more strongly correlated to cell numbers. Based on these, we prefer using both tests in our further experiments.

**Fig. 5:**
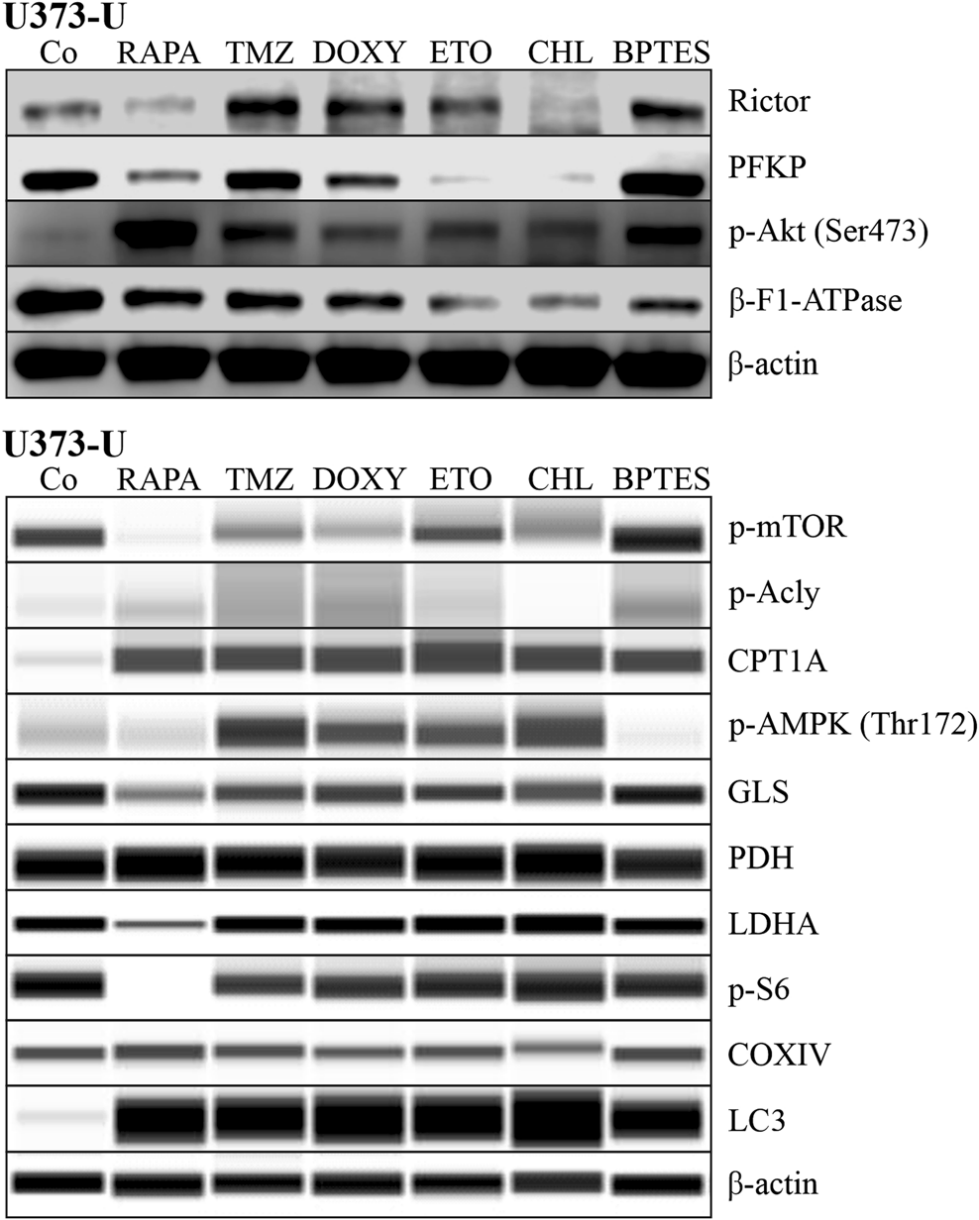
Alteration in metabolic activity related enzyme and protein expressions. The signs of metabolic shifts were analysed after different drug treatments (RAPA – rapamycin 50 ng/ml; TMZ – temozolomide 100 μM; DOXY – doxycycline 10 μM; ETO – etomoxir 50 μM; CHL – chloroquine 50 μM; BPTES - bis-2-(5-phenylacetoamido-1,3,4- thiadiazol-2-yl)-ethyl sulfide 10 μM were applied for 72 h) in U373-U cell line by Western blot (upper image) or WES Simple capillary immunoassay system (lower image)–(the predicted protein sizes were shown in Table 1

Based on its highly resistant phenotype, U373-U cell line was selected for further analysis. To study the alterations of the metabolic enzyme expression quantitatively WES Simple capillary immunoassay and/or Western blot technique were applied. In this cell line, TMZ, DOXY, ETO and BPTES have no significant effect on the proliferation, however, RAPA and CHL have certain anti-proliferative effects (Fig. 3). In almost every single drug treatment performed on U373-U cells, the metabolic enzyme protein expression patterns (Western blot, WES Simple) were rewired. These alterations were mainly the expected ones. In case of TMZ, DOXY, ETO and BPTES treatments Rictor expressions were upregulated and some other potential compensatory mechanisms were activated (Fig. 5). For example, in TMZ treated cells both the mitochondrial (COXIV and β-F1-ATPase expressions) and the glycolytic (PFKP, PDH, LDHA) enzymes were intact; p-AMPK level, CPT1A expression and the related β-oxidation were upregulated in correlation to higher LC3 (activation of autophagy) and lower GLS expressions (no induced glutamine utilisation) and lipid synthesis was blocked by the decreased Acly activation. U373-U cells could find alternative metabolic pathways and could proliferate at the same rate as control cultures in 72-h treatment period after TMZ. In addition, in RAPA or CHL treated cells many unpredictable and conflicting alterations could also occur. As an example, Rictor expressions were slightly downregulated and the sign of some mitochondrial compensatory mechanism could be observed in the analysed Alamar Blue and SRB results after RAPA treatments. However, neither glycolytic nor compensatory, other substrate utilisation pathways could be activated in these cells. GLS, PFKP were downregulated and the expression of other proteins related to potential lipid and autophagy derived compensatory mechanisms were rather conflicting. In the other anti-proliferative CHL treatment, we could not find real compensatory mechanism, glycolysis, glutaminolysis or mTOR could not be activated. This could be the sign of metabolic failure or catastrophe. In addition, to understand such a chaos further studies and biochemical functional analyses are needed.

### The In Vitro Effect of Combining Metabolic Inhibitors in Glioma Cells

As we detected lower effects in high-grade glioma cell lines after in vitro mono-drug treatments, we applied certain combined treatments and compared their effects to RAPA+TMZ combination results using both Alamar Blue and SRB tests (Table 3.). However, our results draw attention to certain significant differences observed in the results of Alamar Blue and SRB tests; nevertheless these could only be interpreted in some combinations. The results of CHL combined treatments were the strangest in those cases. It is conceivable that the two inhibited metabolic pathways (including autophagy) try to activate mitochondrial compensatory mechanisms and alter (disturb) FADH + H^+^ concentrations. While ETO + CHL, BPTES+CHL and RAPA+CHL combinations seemed to be less effective according to Alamar Blue test results of U373-U cells. These combinations in correlation to their predictive effect (to block two alternate compensatory mechanisms) were really effective based on the protein content of the remaining cells. It was really surprising that BPTES+ETO and BPTES+DOXY treatments were the less effective combinations in the studied three high-grade glioma cell lines. Moreover, the other tested combinations reduced the proliferation of these high-grade glioma cells to about 50% or lower. The RAPA+CHL combination was really effective in correlation to its altering function in cellular metabolism and regulation of the three glioma cell lines.

**Table 3:** The anti-proliferative effects of combined treatments (Alamar Blue and SRB tests)

Combined treatments		AB		SRB	
U373-U	RAPA + TMZ	76 ± 5.9%	S	44 ± 3.11%	S
	RAPA + DOXY	66 ± 7.9%	S	48 ± 6.19%	S
	RAPA + ETO	70 ± 4.6%	S	44 ± 6.25%	S
	RAPA + CHL	78 ± 4.7%	–	28 ± 5.15%	A
	BPTES + ETO	97 ± 10.4%	–	85 ± 4.18%	S
	BPTES + CHL	80 ± 4.3%	A	44 ± 3.9%	S
	BPTES + DOXY	83 ± 7.6%	S	94 ± 6.1%	–
	DOXY + CHL	69 ± 2.4%	S	51 ± 1.29%	S
	CHL + ETO	82 ± 3.8%	S	48 ± 6.5%	S
U87	RAPA + TMZ	65 ± 9.4%	S	48 ± 7%	S
	RAPA + DOXY	56 ± 8.2%	S	55 ± 3.1%	S
	RAPA + ETO	65 ± 8.3%	–	56 ± 2%	S
	RAPA + CHL	52 ± 7.5%	S	38 ± 5.4%	A
	BPTES + ETO	79 ± 13.3%	S	86 ± 1.8%	–
	BPTES + CHL	55 ± 2.4%	S	45 ± 0.6%	–
	BPTES + DOXY	67 ± 11.1%	–	83 ± 1.6%	–
	DOXY + CHL	57 ± 3.5%	A	40 ± 0.4%	S
	CHL + ETO	82 ± 13.1%	–	48 ± 0.9%	S
U251	RAPA + TMZ	65 ± 7.1%	S	48 ± 4%	A
	RAPA + DOXY	56 ± 9%	A	55 ± 2.5%	S
	RAPA + ETO	65 ± 6.6%	A	56 ± 1.9%	S
	RAPA + CHL	52 ± 4.7%	–	38 ± 1.9%	A
	BPTES + ETO	79 ± 4.3%	–	86 ± 5.9%	–
	BPTES + CHL	55 ± 4.2%	A	45 ± 2.4%	A
	BPTES + DOXY	67 ± 4.6%	–	83 ± 3.8%	–
	DOXY + CHL	54 ± 6.6%	–	48 ± 2.4%	S
	CHL + ETO	65 ± 3.2%	–	47 ± 3.1%	S

The detected proliferation rates were given relative to controls (%). The additive (A) or the synergistic (S) effects of different drug combinations (RAPA – rapamycin 50 ng/ml; TMZ –temozolomide 100 μM; DOXY – doxycycline 10 μM; ETO – etomoxir 50 μM; CHL – chloroquine 50 μM; BPTES – bis-2-(5-phenylacetoamido-1,3,4- thiadiazol-2-yl)-ethyl sulfide 10 μM) were calculated using the data sets of parallel monotherapies

## Discussion

The standard treatments surgery, radio- and chemotherapy – dominantly – have many limitations in GBM patients [29], the most important ones are the following: a. the special site of the body – the blood-brain barrier filters many drugs; b. the tissue and genetic heterogeneity of the tumours; c. high resistance rate; and d. toxic side-effects (main targeted processes are also active in normal/non-malignant proliferating cells, as well). The high relapse rate is in good correlation to the first three points, moreover, quiescent tumour cells (e.g. cancer stem cells/dormant cells/glioma stem cells) may escape from the conventional therapies with many different strategies [30]. In 2011, Hanahan and Weinberg gave additional fundamental cancer hallmarks to explain these strategies and the complexity of tumorigenesis and tumour evolution. Nowadays, metabolic rewiring is an emerging hallmark in cancer research. The recently highlighted metabolic shifts support the rapid proliferation or the survival of highly resistant tumour cells in toxic microenvironment in order to facilitate energy production, macromolecule synthesis and maintenance of redox homeostasis [31].

It was clearly demonstrated in different studies that cancer bioenergetics is changed with dynamic alterations at metabolic level [13]. In our previous work, we tested TMZ with combined metabolic inhibitors, however, glutaminase inhibitors have not been studied [22]. It is well-known that beside glucose other important substrates can fuel bioenergetic mechanisms in mammalian cells. In the present study, we compared the effects of glutaminase inhibitor (BPTES) or RAPA combining with other metabolic inhibitors; including RAPA+TMZ treatment effectivity.

The connection between glutaminolysis and glycolysis, their dependence on cellular mTOR activity [32] were deeply investigated in many previous glioma studies [31, 33]. In addition, both mTOR complexes have been described to play an important role in the regulation of these processes. In our recent study, we could underline the importance of individual differences and metabolic alterations in therapeutic failures, especially the enhanced Rictor expressions after different treatments (TMZ, DOXY, ETO, BPTES). Based on these, mTORC2 driven metabolic shift in correlation to the detected AMPK and autophagy activation could suggest combining metabolic targets using dual mTOR inhibitors or combining the previous drugs with mTOR inhibitory treatments [31, 34, 35].

Our another interesting observation is the shift to mitochondrial oxidative phosphorylation after several mono- or combined treatments (e.g. in RAPA and CHL or RAPA+CHL, respectively) which can be assigned from the different results of Alamar Blue and SRB tests. This metabolic alteration towards OXPHOS can be targeted with different agents. This finding confirms that OXPHOS co-targeting could be another good option in the future as it has been suggested in Gboxin therapy or other antibiotics targeting mitochondrial functions (e.g. doxycycline) [36, 37].

According to our findings, the best combination strategies seemed to be the different RAPA combinations (TMZ/DOXY/ETO/CHL), except for RAPA+BPTES, which underline the importance of multi-targeting metabolic pathways.

RAPA has more targets, it could inhibit glycolysis, glutaminolysis and several kinase activities related to other cellular mechanisms than BPTES – glutaminase inhibitor. RAPA plus one additional metabolic pathway targeting inhibitor could have more success than glutaminase inhibitor combinations if the side-effects will not increase in the patients.

Many publications have reported that using autophagy inhibitors in combination with targeted kinase inhibitors could have more success in gliomas, as well [38]. It was described that high-grade gliomas have high LC3 autophagy marker expression in tissue microenvironment, since gliomas use autophagy as a survival mechanism frequently [39]. Our results confirm that autophagy inhibitors could enhance the effect of many different anti-metabolic drugs. RAPA+CHL and RAPA+ETO were the most effective ones, these combinations had extremely high anti-proliferative effect, and these could even induce metabolic catastrophe in all high-grade glioma cells. In these cases, mTOR inhibitory effects were supplemented with blocking another alternative catabolic energy source related to autophagy/lipid oxidation. BPTES and DOXY could not have such a great effect in our 72-h treatments in concordance with the glutamine oxidation pathway – these two inhibitors involved in the same route in cellular metabolism and energy production [40].

Finally, our data suggest that the detected metabolic heterogeneity (the high mTORC2 complex activity, enhanced expression of Rictor, p-Akt, p-S6, CPT1A and LDHA enzymes in glioma cases) is a very promising combination target. This suggest studying the in vivo effect and the use of many different new or already known drugs with less potential side-effects in the future therapy of glioma models. However, further studies are needed to find the best combinations for patients. Therefore, it should be considered to map tissue heterogeneity and alterations with several cellular metabolism markers in biopsy materials after applying recently available or new treatments.
